# Association between e-cigarette use and parents’ report of attention deficit hyperactivity disorder among US youth

**DOI:** 10.18332/tid/136031

**Published:** 2021-06-04

**Authors:** Bekir Kaplan, Arik V. Marcell, Tugba Kaplan, Joanna E. Cohen

**Affiliations:** 1 Institute for Global Tobacco Control, Johns Hopkins Bloomberg School of Public Health, Baltimore, United States; 2 Department of Pediatrics, Johns Hopkins Medicine, Baltimore, United States; 3 Population, Family and Reproductive Health, Johns Hopkins Bloomberg School of Public Health, Baltimore, United States; 4 Department of Medicine, Anne Arundel Medical Center, Annapolis, United States

**Keywords:** adolescent, electronic nicotine delivery product, attention deficit disorders

## Abstract

**INTRODUCTION:**

There is paucity of literature that evaluates e-cigarette use rates among the youth with attention deficit hyperactivity disorder (ADHD). The aim of this study is to compare the rates of cigarette only, e-cigarette only, dual use, and initiation age of regular use and trying to quit cigarettes or e-cigarettes among the youth with and without ADHD.

**METHODS:**

We used Population Assessment of Tobacco and Health (PATH) study Wave 3 (2015–2016) youth data, a nationally representative cross-sectional study in the US. The main outcome was tobacco use status of youth and ADHD diagnosis was based on parent report.

**RESULTS:**

The survey included 11801 youth (50%, 12–14 years; 49% female). Compared to youth without ADHD, the relative risk ratio (RRR) was 1.79 (95% CI: 1.02–3.21) for cigarette only use, 1.41 (95% CI: 1.01–2.21) for e-cigarette only use, 3.40 (95% CI: 1.69–6.84) for dual use, 1.75 (95% CI: 0.92–3.35) for cigarette and other product(s) use, 1.48 (95% CI: 0.58–3.77) for e-cigarette and other product(s) use, and 3.37 (95% CI: 1.88–6.17) for poly use among youth with ADHD, after adjusting for age group, sex, and race/ethnicity.

**CONCLUSIONS:**

Cigarette only use, e-cigarette only use, dual use of cigarettes and e-cigarettes, and poly use of cigarettes, e-cigarettes, and other product(s) were significantly associated with parent report of an ADHD diagnosis. It is critical for healthcare providers to be screening youth for e-cigarette use, especially youth who are diagnosed with ADHD.

## INTRODUCTION

E-cigarette use in the US has increased dramatically and exceeded use of combustible cigarettes among youth in recent years^1^. In the US, 19.6% of high school students (3.02 million) and 4.7% of middle school students (0.55 million) currently used e-cigarettes every day or somedays in 2020^2^. Most e-cigarettes contain nicotine and results from prior cross-sectional analysis show that e-cigarette use is associated with increased risk for cigarette initiation^3^. Past longitudinal and cross-sectional research shows that people with mental health conditions (MHC) are more likely to smoke cigarettes compared to those without MHCs^4^. This is of particular concern among youth with attention deficit hyperactivity disorder (ADHD) – one of the most prevalent mental health disorders of childhood^5,6^ – since these youth may self-medicate with nicotine products^7^ to regulate negative affect associated with attentional dysfunction and improve their attention^8^.

It has been well documented that adolescents with ADHD smoke more cigarettes compared to those without ADHD^9-13^. Moreover, earlier initiation of cigarette smoking^14^, quicker progression to regular cigarette use^15^, more severe nicotine dependence^9^, and more difficulty quitting smoking^16^ have been reported among people with ADHD. In addition, early cigarette use may increase the likelihood for subsequent substance use disorders in ADHD individuals^17^. To date, very little research has assessed the association between ADHD and e-cigarette use among youth^18,19^. In a one-year longitudinal study of ninth grade students in California, ADHD symptomology was associated with increased risk of e-cigarette initiation^18^. Another longitudinal study of high school seniors from four high schools in the US mid-Atlantic region reported ADHD symptoms in high school predicted increased risk for e-cigarette use in college^19^. To our knowledge, the association between ADHD and e-cigarette use among youth has not been evaluated using nationally representative US data. This is especially relevant since e-cigarette use in the US has increased dramatically and exceeded use of combustible cigarettes among youth in recent years^1^ and nearly 40% of all adolescents have low levels of ADHD symptomatology^5,6^. Exploring the association between ADHD and e-cigarette initiation age, and e-cigarette quit attempts may help to understand the increase in e-cigarette use among youth.

To address gaps in the current literature, the main aim of this study was to compare the rates of cigarette use only, e-cigarette use only, and dual use of cigarettes and e-cigarette, among youth (aged 12– 17 years) with and without ADHD, using nationally representative data. We also explored the initiation age of regular use and trying to quit cigarettes or e-cigarettes among youth with and without an ADHD diagnosis. Such analyses are critical given that childhood externalizing disorders, primarily ADHD, have been defined as strongly predictive of nicotine use and dependence in the National Comorbidity Survey Replication^20^.

## METHODS

### Study procedures

This study uses publicly available youth data from wave 3 (October 2015–2016) of the Population Assessment of Tobacco and Health (PATH) Study, a nationally representative, longitudinal study of more than 46000 adults and youth participants in the US, using a four-stage stratified area probability sample design^21^. Data were collected using computer-assisted personal interviewing for screener and parent interviews, and audio computer assisted self-interviewing for adult and youth interviews. The weighted retention rate between Wave 1 and Wave 3 was 78.4%. PATH collects information from youth and adults about use of various tobacco products including cigarettes and e-cigarettes, tobacco dependence, cessation, perceptions of risk and harm, overall physical and mental health, peer and family influences, substance use, and demographic information^22^. Information on the sampling procedures can be found in the PATH User Guide^21^. For these analyses, the analytic sample consisted of 11801 youth aged 12–17 years. Since the current study is a secondary data analysis of existing data, it is exempt from approval by the Institutional Review Board of the Johns Hopkins Bloomberg School of Public Health.

### Measures

#### Current cigarette and e-cigarette use

In the PATH Study youth data, ‘current use’ of any tobacco product was defined as using a tobacco product within the past 30 days. If a participant used at least one other tobacco product (i.e. cigar, hookah, smokeless, snus, kretek) within the past 30 days, we coded them as ‘other tobacco product users’ (yes/no). A combined measure was created that categorized participants as follows: 1) dual users of cigarettes and e-cigarettes; 2) cigarette only users; 3) e-cigarette only users; 4) poly users (cigarette and e-cigarette users who use at least one other product); 5) cigarette and other product users; 6) e-cigarette and other product users; and 7) non-users of any tobacco product.

#### Assessment of ADHD

Parents of the youth were asked about their youth’s ADHD history with the following question: ‘In the past 12 months, has your child been told by a doctor, nurse or other health professional that (he/she) has ADHD?’. A follow-up question was asked to the parent of new baseline youth respondents who were not told they had ADHD in the past year: ‘Has your child ever been told by a doctor, nurse or other health professional that (he/she) has ADHD or ADD?’. We coded ‘ever ADHD diagnosis’ as ‘yes/no’.

#### Tobacco initiation age and quit attempts

Tobacco initiation age was assessed by asking respondents the age ranges when they first started smoking cigarettes or e-cigarette use regularly with options ‘aged 12–14 years’ and ‘aged 15–17 years’. Cigarette quit attempts for cigarette only users was assessed by ‘In the past 12 months, have you tried to completely stop smoking cigarettes?’ and e-cigarette quit attempts for e-cigarette only users was assessed by ‘Have you tried to completely stop using [e-cigarette] within the past 12 months?’; both questions were coded as ‘yes/no’.

#### Covariates

Participants were also assessed on their age (coded as 12–14 years or 15–17 years); sex (coded as male; female); and race/ethnicity (coded as non-Hispanic white; non-Hispanic black; Hispanic; and others).

### Statistical analysis

All analyses were performed using STATA version 15.1 (Statacorp., College Station, TX). The PATH Study population weights were used to adjust for the complex study design including oversampling and non-response. Fay’s method, a variant of balanced repeated replication method, was used to form replicative weights in variance estimation in all analyses. The Fay coefficient was specified at 0.30, as recommended by the PATH Study^20^. We present unweighted frequencies and weighted percentages in the tables. Further information on the weighting procedure can be obtained from the PATH Study Public-Use Files^17^. Chi-squared, multivariable logistic regression, and multinomial logistic regression were used to examine the association between tobacco product use and ADHD status, controlling for covariates for potential confounding. All tests were two-sided with significance level set at 5%.

## RESULTS

The study population of 11801 youth included 5996 (50.4%) aged 12–14 years, 6095 (48.6%) females, 5336 (53.1%) non-Hispanic Whites, 132 (1.2%) cigarette only users, 243 (2.2%) e-cigarette only users, 87 (0.8%) dual users of both cigarettes and e-cigarettes, 79 (0.7%) cigarette and other product(s) users, 62 (0.6%) e-cigarette and other product(s) users, 62 (0.6%) poly users, and 10755 (93.9%) non-users. The parents of 1240 (10.8%) youth reported their child has ever been told by a health professional that he/she has ADHD (Table 1).

**Table 1:** Sociodemographic characteristics of participants, PATH study wave 3 (2015–2016) youth data, US (N=11801)

*Characteristics*	*n*	*% (95% CI)*
**Age** (years)		
12–14	5996	50.4 (49.9–50.9)
15–17	5805	49.6 (49.1–50.0)
**Sex**		
Male	6095	51.4 (51.2–51.6)
Female	5673	48.6 (48.4–48.8)
**Race/ethnicity**		
Non-Hispanic White	5336	53.1 (52.7–53.5)
Non-Hispanic Black	1518	13.1 (12.8–12.4)
Hispanic	3438	23.8 (23.5–24.2)
Other	1098	9.9 (9.7–10.3)
**Tobacco use status**		
Cigarette only users	132	1.2 (1.0–1.4)
E-cigarette only users	243	2.2 (1.9–2.6)
Dual users of cigarettes and e-cigarettes	87	0.8 (0.6–0.9)
Cigarette and other product(s) users	79	0.7 (0.5–0.9)
E-cigarette and other product(s) users	62	0.6 (0.5–0.8)
Poly users [cigarettes, e-cigarettes, and other product(s)]	62	0.6 (0.5–0.8)
Non-users	10755	93.9 (93.3–94.5)
**ADHD**		
No	10561	10.8 (10.0–11.5)
Yes	1240	89.2 (88.5–90.0)
**Total**	11801	100.0

ADHD: attention deficit hyperactivity disorder.

Among youth with ADHD, 18 (1.6%) were cigarette only users, 30 (3.0%) e-cigarette only users, 17 (1.7%) dual users, 12 (1.0%) cigarette and other product(s) users, 8 (0.8%) e-cigarette and other product(s) users, 20 (1.6%) poly users, and 1070 (90.3%) non-users. Among youth without ADHD, 114 (1.1%) were cigarette only users, 213 (2.1%) e-cigarette only users, 70 (0.7%) dual users, 67 (0.7%) cigarette and other product users, 54 (0.6%) e-cigarette and other product(s) users, 42 (0.5%) poly users, and 9685 (94.3%) non-users (p<0.001) (Table 2).

**Table 2:** Tobacco use status of youth with and without ADHD and multinomial logistic regression model of being a tobacco user among youth with ADHD compared to youth without ADHD, PATH Study Wave 3 (2015–2016) Youth Data, US (N=10755)

*Tobacco use status*	*ADHD*				p*	*RRR ^a^ (95% CI)*
	*Yes*		*No*			
	*n*	*%*	*n*	*%*		
Cigarette only users	18	1.6	114	1.1		**1.79 (1.02–3.21)**
E-cigarette only users	30	3.0	213	2.1		**1.41 (1.01–2.21)**
Dual users of cigarettes and e-cigarettes	17	1.7	70	0.7		**3.40 (1.69–6.84)**
Cigarette and other product(s) users	12	1.0	67	0.7	<0.001	1.75 (0.92–3.35)
E-cigarette and other product(s) users	8	0.8	54	0.6		1.48 (0.58–3.77)
Poly users [cigarettes, e-cigarettes, and other product(s)]	20	1.6	42	0.5		**3.37 (1.86–6.11)**
Non-users	1070	90.3	9685	94.3		1

ADHD: attention deficit hyperactivity disorder.

* Chi-squared test.

a Relative Risk Ratio: RRR was adjusted by age, sex, and race/ethnicity.

After adjustment for age, sex, and race/ethnicity, compared to youth without ADHD, the relative risk ratio (RRR) was 1.79 (95% CI: 1.02–3.21) for cigarette only use, 1.41 (95% CI: 1.01–2.21) for e-cigarette only use, 3.40 (95% CI: 1.69–6.84) for dual use, 1.75 (95% CI: 0.92–3.35) for cigarette and other product(s) use, 1.48 (95% CI: 0.58–3.77) for e-cigarette and other product(s) use, and 3.37 (95% CI: 1.88–6.17) for poly use among youth with ADHD (Table 2).

Among youth with ADHD, ever being a regular user of cigarettes and e-cigarettes before the age of 14 years was reported by 62.1% and 51.1% of youth, respectively; and 54.3% and 42.1% among youth without ADHD, respectively (p=0.492 and p=0.428). Among youth with ADHD and who currently used cigarettes or e-cigarettes, trying to quit cigarettes and e-cigarettes in the last 12 months was reported by 37.5% and 28.8%, respectively, and by 40.7% and 28.3% of youth without ADHD, respectively (p=0.736 and p=0.934) (data not shown).

With regard to ADHD prevalence, 13.7% of cigarette only users, 14.3% of e-cigarette only users, 23.7% of dual users of cigarettes and e-cigarettes, 15.6% of cigarette and other product(s) users, 14.0% of e-cigarette and other product(s) users, 26.7% of poly users, and 10.1% of non-users had ADHD diagnosis (p<0.001) (data not shown).

## DISCUSSION

This is the first nationally representative study in the US to report higher e-cigarette use rates among youth with parent-reported ADHD compared to youth without ADHD. The e-cigarette use rate among youth with parent-reported ADHD was almost 50% higher than among youth without ADHD. In addition, poly use and dual use of both cigarettes and e-cigarettes were also higher among youth with ADHD compared to youth without ADHD. The association between ADHD and combustible cigarette use is well-established. This study extends this association to e-cigarettes that are very popular among today’s youth in the US. The association between poly tobacco use and ADHD was also reported previously^18^, but not using nationally representative data. To the best of our knowledge, this is the first nationally representative study to report association between poly tobacco use and ADHD among youth. Consistent with the National Comorbidity Survey Replication study^20^, higher rates of e-cigarette use among youth whose parents report that they were diagnosed with ADHD is concerning. It is critical for healthcare providers to be screening youth for e-cigarette use, especially youth who are diagnosed with ADHD.

The mechanism of the association between ADHD and e-cigarette use still needs to be better understood. Several factors may contribute to the association observed in the current study. ADHD is characterized by difficulty in attention, impulsivity, and poor decision making^5^. All of these features and youth perceptions of e-cigarettes being less harmful than cigarettes^23^ may lead to higher e-cigarette use rates among youth with ADHD. In addition, in order to control their attentional dysfunction, some youth with ADHD may be self-medicating to alleviate their symptoms of ADHD by using nicotine^7^. It has been hypothesized that the greater incidence of smoking might reflect an attempt at self-medication of ADHD symptoms^24^ and indirect evidence from adults suggests that nicotine treatment can attenuate dysfunction among those with ADHD^25,26^. This theory, however, has not been sufficiently assessed among youth and the effect of tobacco use on ADHD symptoms among youth has not been studied thus far. Hence, it is not known whether tobacco use deteriorates or alleviates ADHD symptoms especially among youth.

ADHD is a risk factor for early onset of combustible tobacco use^14^. In the current study, initiating regular use of e-cigarettes before the age of 14 years was almost 10% higher among youth with ADHD compared to youth without ADHD. This difference was not statistically significant, likely because of the low sample size; however, this outcome raises the concern that ADHD could also be a risk factor for early onset of e-cigarette use among youth in the US.

It is well known that people with ADHD have more difficulty quitting smoking^17^. In this study, the proportions of youth trying to quit e-cigarettes in the last 12 months were identical between youth with and without ADHD, consistent with the Pittsburgh ADHD Longitudinal Study (PALS) of youth and young adults^27^. However, young age, short usage time and small number of quit attempts of youth compared to adults could be an explanation of this result. In addition, given the low sample size in this study, more research is needed to understand the association between ADHD diagnosis and e-cigarette quit attempts among youth.

ADHD treatment has been found effective in decreasing the risk for smoking among youth^28^ but has not been found to be effective in improving smoking cessation success in adults^29^. Hence, treating ADHD during childhood or adolescence may be critical to help protect people with ADHD from becoming cigarette smokers in adulthood.

### Limitations

This study has several limitations. First, the association between ADHD and tobacco use is cross-sectional. Therefore, it does not address the causal association between ADHD and e-cigarette use among youth. Second, the ADHD diagnosis relied on parents’ self-reports, though parents may be a more reliable reporter of their youth’s health condition compared to their youth^30^. This is different from a number of studies examining ADHD and nicotine, which used a thorough diagnostic assessment of ADHD following DSM criteria. We do not know what (if any) diagnostic criteria were used by respondents, and it is likely that there are false positive and false negative cases. Other factors that were not assessed in the PATH study may also be associated with youth having ADHD symptoms, such as health insurance status. Third, we were not able to assess youth who had clinically non-significant ADHD symptoms; ENDS use rates might be higher among these youth compared to youth without ADHD symptoms. Fourth, we were unable to control for health insurance status of participants because having health insurance was not assessed by the PATH Study. Fifth, it is possible that internalizing/ externalizing problems may be related to e-cigarette use. Future work should consider examining these conditions while assessing the association between ADHD and e-cigarette use. Fifth, the information of tobacco use status of the participants relied on self-reported information and did not include biological verification of nicotine product use. Finally, there was a limited number of participants for assessing quit rates, initiation age for tobacco use, and taking ADHD medication regularly. Despite these limitations, relying on nationally representative data is a strength of this study.

## CONCLUSIONS

This is the first nationally representative study to report that e-cigarette only use, dual use of both cigarettes and e-cigarettes, and poly use were significantly associated with parent report of an ADHD diagnosis among youth. Educating parents and youth about e-cigarettes, especially those with ADHD, may help to curb e-cigarette use among youth in the US. Further, it is critical for healthcare providers to be screening youth for e-cigarette use, especially youth who are diagnosed with ADHD. Further examination on the association between dual and poly tobacco use and ADHD is warranted, given the evolving landscape of available products and the paucity of published information on the health effects associated with different combinations of tobacco use.
